# Examining the development of PTSD symptoms in individuals who witness acute stress reaction on the battlefield

**DOI:** 10.1192/bjo.2021.25

**Published:** 2021-04-01

**Authors:** Judith Harbertson, Lauretta Ziajko, Jessica Watrous

**Affiliations:** School of Public Health, San Diego State University, USA; Department of Psychiatry, Uniformed Services University of Health Sciences, USA; Psychiatry Residency Program, Naval Medical Center (San Diego), USA; Leidos, Inc., San Diego, USA

**Keywords:** Acute stress disorder, post-traumatic stress disorder, military personnel, deployment, combat disorders

## Abstract

Adler et al describe an innovative perspective on battlefield posttraumatic stress disorder (PTSD) symptoms in response to an acute stress reaction (ASR), tracking not the individual experiencing ASR, but rather the service members who witness another team member experiencing an ASR. PTSD symptoms, reactions, observations and responses in the witness are assessed.

By examining post-traumatic stress disorder (PTSD) symptoms among those who witness another individual experiencing an acute stress reaction (ASR) on the battlefield, Adler et al^1^ create a unique and innovative perspective transitioning our focus in battlefield medical care beyond the individual who experiences an ASR to the witness of the ASR. Given the nature of war, experiencing trauma is a significant possibility for those exposed to combat. Although much attention has been paid to management of acute physical injuries on the battlefield (e.g. research aimed at improving field medical care, improvements in air evacuation), less is known about managing the immediate effects of acute psychological injury and the variety of individuals this may affect. Adler et al challenges us to rethink how military personnel respond to others’ acute psychological injuries, how their training and response may affect their development of PTSD symptoms, and what could be done to improve management of these responses.

In the study, Adler et al assessed two groups of USA army service members: one group who had been previously deployed (*n* = 176) and a second group that was currently deployed (*n* = 497). They screened individuals for PTSD (PTSD Checklist-5), ASR and combat exposure, and, importantly, if they witnessed another person experiencing an ASR and how they responded to this individual (such as providing instruction or being unsure how to respond). The three primary goals of these analyses were to determine the prevalence of individuals who witness an ASR during combat; examine whether witnessing an ASR was correlated with screening positive for PTSD/subthreshold PTSD in the individual who witnessed the ASR in another individual; and describe the range of responses observed in the individual experiencing the ASR, and interventions provided by the witness. Compared with the currently deployed sample, the previously deployed sample was slightly older in age, higher ranking, reported more combat experiences, witnessed a higher proportion of ASRs and a lower proportion screened positive for any form of PTSD.

Adler et al reported that 42–52% of the two groups witnessed one or more service members experience an ASR. In adjusted analysis, reporting high frequency of witnessing an ASR was associated with PTSD/subthreshold PTSD in the previously deployed sample (odds ratio 8.7, 95% CI 2.3–42.6) and approached significance in the currently deployed sample (odds ratio 1.7, 95% CI 0.9–2.8). Being unable to function and appearing detached were the most common reactions observed by the witness, which can be detrimental to both the individual and the team in an active combat environment; 31% of witnesses reported the person experiencing an ASR was a risk to themselves and the team. Common interventions provided by the individual who witnessed the ASR were trying to calm the team member down, providing directive to perform a simple task and eliciting help from others.

Although the previously deployed sample reported more incidents of witnessing ASRs, they had a lower proportion who screened positive for PTSD. However, the association between witnessing an ASR and screening positive for PTSD/subthreshold PTSD in adjusted analysis remained statistically significant. Among the previously deployed sample, a high level of witnessing ASRs was strongly associated with screening positive for PTSD; in the currently deployed population that had more recently witnessed an ASR, it was only approaching a significant association, possibly suggesting that not enough time had passed for PTSD symptoms to fully manifest. However, there was a substantially higher proportion of subthreshold PTSD among the currently deployed sample (19 *v*. 7%) than the previously deployed sample, possibly suggesting that subthreshold PTSD is an early psychological consequence of ASR exposure, whereas PTSD is a long-term psychological consequence of ASR exposure. The previously deployed sample differed from the currently deployed sample in a few key ways. The previously deployed sample was older, higher ranking and reported more combat experiences. The currently deployed sample was younger and lower ranking (i.e. enlisted service members may have different combat experiences to officers), with fewer combat experiences and higher proportions of subthreshold PTSD. As they age, increase in rank and re-deploy, a subset of service members may separate from the military, and those remaining may have a lower proportion of PTSD compared with the separated service members. Alternatively, those with years of experience in combat deployments may respond differently to a crisis than those with little experience. Combat experience may allow individuals to develop resilience against PTSD, particularly for those with a low level of witnessing ASRs; although this may not affect the association between witnessing high levels of ASR and screening positive for PTSD.

An explanation for why the currently deployed sample had higher rates of subthreshold PTSD symptoms could be that when deployed, some stress reactions (like hypervigilance for anything in the roadway that could be an improvised explosive device) may be adaptive. However, back home in a non-combat environment, such reactions are often maladaptive. Therefore, although fewer PTSD symptoms in the previously deployed sample may have resulted from individuals with more severe symptoms separating from the military and no longer being a part of this cohort, or from individuals with low levels of witnessing ASRs developing resilience over time with increased combat experience, it is possible that individuals in the currently deployed sample are exhibiting behaviours that look similar to PTSD symptoms, but are adaptive and protective in the deployed environment, and may serve as a confounder/source of misclassification. Additionally, it is important to note that this study was cross-sectional, and causality could not be established. Timing between witnessing an ASR and PTSD symptoms was also not assessed. It is possible that some service members who witnessed an ASR recently had not yet reported symptoms (in the currently deployed sample) but would develop them subsequently, or if service members in the previously deployed sample who witnessed an ASR had already received treatment and their PTSD symptoms improved or resolved. This could result in fewer individuals screening positive for PTSD symptoms, and the study's findings could reflect an underestimate of the effect size or significance of the association between witnessing ASRs and ever developing PTSD symptoms.

In adjusted analysis, combat experiences (five or more) was the only variable significantly associated with PTSD/subthreshold PTSD in the currently deployed sample, which may indicate that the association of ASR with PTSD in the previously deployed sample may have been reinforced by their recall of past combat events. Adler et al suggests that the significant association between ASR and PTSD in the previously deployed sample, and marginal significance in the currently deployed sample, could be related to ‘anticipatory anxiety’ for the upcoming deployment that may elicit memories of witnessing an ASR in previous combat and reinforce PTSD symptoms. However, it is important to note that the study did not collect information on whether the individual who witnessed another person having an ASR ever experienced an ASR themselves, during the current deployment or prior, which the study authors acknowledge. This raises question of whether in the currently deployed sample, the association between witnessing another experiencing an ASR and PTSD is confounded by prior ASRs that the individual experienced. Prior combat experience was significantly associated with PTSD/subthreshold PTSD in the currently deployed sample, which could be a proxy for the individual experiencing an ASR in past combat. It is then possible that a prior ASR in the individual is driving the association with PTSD/subthreshold PTSD, if the proportion of individuals with a prior ASR was not weighted evenly between the group that had witnessed another experiencing an ASR and the group that had not. Likewise, in the previously deployed sample, the effect size of the association between high levels of witnessing ASRs and PTSD/subthreshold PTSD was large. Although the study authors acknowledge that this large effect size may have been inflated owing to the small sample size, it may have also been a proxy for the individual being more likely to experience an ASR themselves when they were repeatedly witnessing ASRs in others. Because these data were not collected, it is impossible to discuss whether the data support this theory or not. Future studies should collect information on whether the individual experienced an ASR previously or when witnessing others experience an ASR, to control for this possibility. These data could inform whether training/interventions need to include a component on managing an ASR in someone else when the witness themselves had experienced one in the past, or if they experienced one when witnessing another service member's ASR.

Before the examination of PTSD development among individuals who witness another individual experiencing an ASR within the military setting, several studies reported higher proportions of PTSD, depression and anxiety symptoms, and antisocial behaviour, among those who directly witnessed a violent murder, emergency resuscitation of a family member, community violence or substance misuse.^2–5^ However, it was unknown whether an individual could develop PTSD from witnessing another individual's reaction (i.e. ASR) to that specific trauma. These data suggest the potential for a shared mechanism for the development of PTSD or other mental health condition among those who witness an individual experiencing trauma and those who witness another individual experience an ASR to a trauma, regardless of whether the setting is civilian or military. Findings from Adler et al, in combination with previous research among those that witness physical violence or self-harm, has contemporary relevance to a substantial civilian population that could be at risk of adverse mental health symptoms resulting from their occupational or personal role (e.g. medical professionals, firefighters, police officers, children/adults who witness domestic violence) witnessing individuals experience an ASR to severe psychological or physical injuries in the community.

Current Veterans Affairs/Department of Defense clinical guidance^6^ on the management of ASR and acute stress disorder (ASD; also referred to as combat and operational stress reaction, if it occurs within a military operational setting) recommend addressing the immediate needs (e.g. safety, food, sleep, medical care, stabilisation) of the ‘person exposed to trauma’ or individual who has experienced the ASR/ASD. This recommendation could also then apply to individuals who witness an ASR if they meet the DSM-5^7^ criteria for experiencing an ASR/ASD themselves.

Within the USA military battlefield setting, there are widely accepted methods of ASR management largely based on guidance formed from data collected among a small sample of soldiers within the Israel Defense Forces (IDF) in 1982, during the Lebanon War.^8^ Treatment programmes based on variations of these principles are referred to as front-line psychiatry,^9^ combat operational stress control,^10^ PIE (proximity, immediacy, expectancy), PIES (proximity, immediacy, expectancy, simplicity)^11^ and BICEPS (brevity, immediacy, centrality, expectancy, proximity).^12^ The study by Solomon and Benbenishty examined PIE among the IDF, specifically, the ‘proximity’ of care to the site where trauma occurred, how quickly the soldier was treated (‘immediacy’) and whether the goals of care (‘expectations’) were clear to the soldier. The treatment included managing basic needs (e.g. sleep, food, time away from combat zone) and allowing the soldier to discuss the traumatic experience (minimal psychiatric intervention). They analysed whether there was an association between PIE components and subsequent development of PTSD symptoms 1 year later among IDF experiencing an ASR. This study found that the lowest levels of PTSD symptoms were reported among soldiers who received all three components of care (i.e. close proximity, immediate care and an understanding that the goal of the treatment was to return them to their unit), with a reduced but protective effect even if they received only one of those components.^8^ A follow-up study^13^ conducted 20 years later among these same soldiers showed sustainability of this finding, with a lower proportion of PTSD symptoms, loneliness and higher social functioning among those who had received one or more components of care at the time of the original event. However, the sample size was small, and despite the numerical difference, there was no statistically significant difference in PTSD symptoms and some other measures (e.g. avoidance score, global distress, occupational functioning) between those who received the components and those who did not.

In 2017, Russell and Figley published a systematic review^9^ examining all data available on mental health outcomes between service members experiencing psychiatric causalities who received front-line care and those who were evacuated. This review showed that, among USA military service members who experienced psychiatric casualties in theatre, there was insufficient evidence to support that current treatment approaches reduced the long-term development of PTSD and other mental health outcomes. Only a few studies^13,14^ examining brief forms of therapy among USA soldiers and marines experiencing psychiatric casualties deployed to Iraq or Afghanistan have been identified. Unfortunately, these studies had substantial study design limitations (e.g. small sample size, no control group, insufficient follow-up time) or did not detect a decrease in long-term health outcomes (e.g. depression, PTSD, substance use) resulting from the intervention. Additional research is needed to rigorously examine treatment approaches and track long-term mental health outcomes among USA military service members in theater, which can then inform evidence-based approaches to care and military mental health policies.

Prior research provides recommendations on how caregivers can provide ‘psychological first aid’ to individuals experiencing a crisis, and the study by Adler et al argues these types of interventions may need to be extended to those who witness individuals experiencing the crisis. Such interventions, when used with individuals experiencing a crisis, suggest a reduction in subsequent morbidity related to the psychological injury.^15,16^ Future studies need to examine whether interventions among witnesses of an ASR could lead to positive outcomes for them as well. These interventions are designed to promote psychological safety, calm and connectedness^17^ by attending to immediate needs (e.g. survival, safety, food, clothing, sleep) and stabilisation, and securing support from the community, friends and family.^18^ At the current time, there is insufficient data to support using trauma-focused psychotherapy or pharmacotherapy in the brief window immediately after the psychological injury among individuals not diagnosed with ASD.^18^ The principles recommended by current Veterans Affairs/Department of Defense guidance (attention to immediate needs, promoting psychological safety, stabilisation, securing support from family and friends) could be examined in both military and civilian populations and extended to those who witness others having severe psychological or physical injuries, if shown to be feasible and effective in preventing or treating symptoms in such witnesses.

Given that the study by Adler et al highlights a new focus of studying PTSD symptoms and ASR responses in witnesses of the experience, much is left to be explored. Future researchers should consider the recommendations below, which include and build upon the study authors’ suggestions for subsequent research.

- Examine the association between witnessing ASRs and PTSD/subthreshold PTSD in other military populations and environments, and at various points across the deployment cycle.
- Create a robust tool to measure witnessing ASR symptoms and possibly other psychological injuries (e.g. brief psychotic episodes, dissociative reactions, depression, bereavement), and include these in combat experience data collection tools because these may be a previously unrecognised cause of PTSD in service members. Validate these tools for widespread use, including semi-structured interviews and systematic observation during military exercises.
- Examine how we identify and care for individuals who witness an ASR.
- Consider how to improve training medics receive to treat service members for psychological injuries on the battlefield, and examine the efficacy of these methods to reduce adverse mental health outcomes through rigorous research studies with adequate follow-up time.
- Develop, study and train service members in structured protocols for actions they can take when they witness an ASR in others. Considerations could include methods that will assist the individual experiencing the ASR, and techniques the witness could use to reduce their chances of developing PTSD symptoms (training discussed in the article is currently being adapted and tested for non-medical unit USA service members, and could be expanded to medics and fellow soldiers likely to witness team members experiencing psychological injuries).
- Adapt ASR management training tools that have already been piloted in the IDF and USA service members,^19–22^ to optimise feasibility and effectiveness among USA service members.
- Explore the association between ASR and PTSD in other occupations and populations that are at high risk for ASR.
- Examine whether guidance on psychological injury interventions used in individuals experiencing the injury are effective in reducing long-term mental health symptoms among those who witness the injury.
- Extend these principles to other civilian medical responders or caregivers who witness severe psychological and physical injury in their occupational or personal community role.

These data by Adler et al suggest that it is time to explore the impact of witnessing ASRs in a variety of at-risk occupational populations; to determine whether there are particular risk factors, as have been shown for other mental health outcomes,^23^ for subsequent PTSD among individuals experiencing or witnessing an ASR; and to train at-risk individuals on how to most effectively intervene,^8,15,17,18^ if doing so is beneficial, when a psychological injury occurs. Data that was unable to be assessed in the study (e.g. presence of a prior or current ASR in the individual, time between witnessing the ASR and report of PTSD symptoms, temporality of the ASR and subsequent PTSD symptoms) could alter conclusions and should be included in future studies to confirm findings. By taking these steps, we may be able to reduce the development of PTSD in those who witness and/or experience ASR or other severe psychological and physical injuries, in both civilian and military communities.
